# Monthly Summary

**Published:** 1879-01

**Authors:** 


### MONTHLY SUMMARY.

*Diffusion of Carcinoma by the Nerves.*—Dr. Calmiatti, who has already published important articles on the pathological histology of the nerves, presents in the *Revista Clinica di Bologna*, an interesting case illustrating his theory of the diffusion of carcinoma by the nerves.

The patient was a man forty years of age, who had previously enjoyed good health, but recently had been attacked with neuralgia of the left cheek, corresponding to the inferior molars. An examination showed that there was a considerable tumor on the inner side of the gum. This tumor, the size of a hen's egg, was removed, and at the same time disarticulation and resection of the jaw as far as the left canine tooth was practised. Though the wound healed readily, the severe neuralgia continued.

The tumor proved to be a carcinoma. It involved the inferior dental nerve, which was followed for more than two centimeters, and into its canal in the bone. There was connected with the tumor and the bone an enlarged and indurated lymphatic gland, belonging to the suprahyoid region. This was also removed. The posterior portion of the nerve, when removed from the canal, was found considerably enlarged.

According to the theories of Robin and Key and Retzius, the nerves contain two species of connective tissue: the *epi-neurium*, and the *peri-neurium*, with its appendix the *endo-neurium*. The epi-neurium connects the bundles which form all nervous trunks, and appears to envelop them; the peri-neurium and endo-neurium form integral parts of each bundle. Their connective tissues consists of small membranes, within which are the so-called peri- and endo-nervous lymphatic spaces, but which have nothing to do with the common lymphatic system. These small membranes which, like the nerve-bundles, maintain a cylindrical form, are arranged in a circular manner at the periphery of the bundles, and are connected and held together from place to place by membranous lamellæ. Hence these peri-nervous lymphatic spaces are multiple and always communicate one with another. The membranes constituting the endo-neurium, which arises from the peri-neurium, pass around each nerve-fibre in such a manner as to form circumscribed endo-nervous lymphatic spaces, which are also continuations of the peri-nervous spaces. The ultimate arrangement of the above-mentioned membrane is such, therefore, that every fibre is suspended in a symphatic space. In the case mentioned we have to take into consideration the lymphatic current which flows in the special canals of the perineurium and the endo-neurium, a current which is in direct communication with the spinal and cerebral subarachnoid spaces, and, by means of these, with the ventricles of the brain, through which the carcinoma cells may pass indefinitely.

The author states that he saw in the section of the dental nerve an abundant infiltration of the carcinoma cells, which were diffused through the peri-nervous and endo-nervous spaces. He also states that this is the second case in which he has found the carcinoma diffused through the inferior dental nerve. The diffusion of the disease was by the nerve solely.—*Lo Sperimentale*, March, 1878.

---

*To Blister the Skin Quickly.*—Into a watch-glass, pill-box, or any similar small receptacle, pour ten drops of concentrated water of ammonia (aqua ammonia fortior); cover the liquid with a bit of linen or a little cotton wool, and at once apply the cup upon the skin where the blister is required. Press so that the vapor is confined to the inside of the vessel. A red circle will directly be observed outside, when it will be certain vesication has taken place. Half a minute or so is all the time required to obtain the result. The blister may be dressed in the usual manner of dealing with a blister from cantharides. Acetic acid, concentrated, applied to the skin will also, in a few minutes, produce vesication. In each case evaporation should be prevented by some suitable covering. Bibulous paper, slightly wetted with a little of the ethereal extract of cantharides, instantly applied to the skin and covered with a piece of adhesive plaster, will answer for the same purpose. —*Ohio Med. Recorder.*

---

*An Early Symptom of Tabes Dorsalis.*—Dr. Berger has a note in the *Centralblatt fur Med. Wis.*, relating to an early symptom of tabes dorsalis, which is described as consisting in the disturbance of the preception of pain of such a kind that stimuli of slight intensity, as well as tactile impressions of a slightful painful kind, such as needle-pricks, are perceived normally; but that more severe and often very strong stimuli produce no greater pain. This analgesia in respect to excessive stimuli may be present before other symptoms of tabes appear, and is of semeiotic importance. This peculiarity, as a common rule, is especially perceived in the skin of the lower extremities, and may finally be observed on the whole cutanous surface, and even on the mucous membrane. It appears to be a symptom depending on an initial lesion of the gray substance.

*Deaths from Anæsthetics.*—The English medical Journals of recent date record a death each, from ether, methylene, and chloroform.

That from ether occurred at the London Hospital, in a patient about to be operated on for hernia. Ether was administered by one of the house-surgeons; the patient was readily brought under its influence. There were no untoward symptoms of any kind; the examination was proceeded with. The man breathed quietly and regularly for a few minutes, and then gave one sudden catching effort at inspiration, and died, in spite of every effort to save him. It is noteworthy that his pulse continued to beat for about thirty seconds after breathing had ceased. At the autopsy the heat was found firmly contracted. There was no strangulation, though the gut showed market traces of having been tightly constricted.

In the death from methylene the victim was a retired officer of 40 years of age, in apparently perfect health. The death was perfectly sudden and inexplicable, and it shows that this anæsthetic is, like chloroform, a poison to some persons of a peculiar idiosyncrasy, and will cause death without any apparent physical reason.—*Med. and Surg. Reporter.*

---

*A New, Cheap and Self-generating Disinfectant.*—Under this title, Dr. John Day, of Geelong, Australia, recommends for use in civil and military hospitals, and also for the purpose of destroying the poison-germs of infectious diseases, a disinfectant composed of one part of rectified oil of turpentine and seven parts of benzine, with the addition of five drops of oil of verbena to each ounce. Its purifying and disinfecting properties are due to the power presumed to be possessed by each of its ingredients, of absorbing atmospheric oxygen, and converting it into either peroxide of hydrogen, or ozone. Articles of clothing, furniture, wall paper, carpeting, books. newspapers, letters, etc., may be perfectly saturated with it without receiving the slightest injury, and when it has been once freely applied to any rough or porous surface, its action will be persistent for an almost indefinite period. This may, at any time, be readily shown by pouring a few drops of a solution of iodide of potassium over the material which has been disinfected, when the peroxide of hydrogen, which is being continually generated within it, will quickly liberate the iodine from its combination with the potassium, and give rise to dark-brown stains. It may be applied with a brush or a sponge, or, if more convenient as is the case with certain articles, such as books, newspapers and letters, it may be simply poured over them until they are well soaked; they may then be allowed to dry, either in a warm room or in the open air.—*New Remedies.*

*Poisoning by Chlorate of Potash.*—A child of Dr. Kauffman, Schuylkill county, Pennsylvania playing doctor with her little brother and sister, found a box of chlorate of potash, of which, after administering various and unknown quantities to her patients, took about half an ounce herself. The child, who was about two and a half years old, commenced vomiting in about an hour, and died in seven hours after taking the medicine. Beside the emetic, also hydro-drastic effects were observed, the child dying of gastritis or inflammation of the stomach and bowels. This case teaches us to be verv careful in using and keeping chlorate of potash. Under no circumstances whatever should it be administered in a crystalline form, as it seems to be in this form a violent irritant to the mucous membrane. The child was given as much water and cream as she could drink, and to the last she vomited crystals of chlorate of potash.—*American Journal of Pharm.*

---

*Warning Against the Hypodermic Use of Morphia.*—Dr. Levinstein, of Berlin, says, in his late work on the "morphia mania," *morphiumsucht*, that it is generally caused by hypodermic injections of the drug, given by the medical attendant during illness, to relieve pain, and continued by the patients themselves after the actual need of it has disappeared, for the sake of the mental excitement it produces. The danger involved was not generally suspected during the first years of the use of the new means of administration, but the existence of the Maison de Sante under the medical direction of the author, which is devoted to the cure of sufferers from this craving, shows how large its evil effects must already be. The sufferers feel quite well for a certain time while using the narcotic, but before long evident symptoms of disease appear. The patients become emaciated, the complexion ashy or dark red, the eyes lack lustre, and vision is often deranged; thirst, nausea, and loss of appetite, constipation, and the secretion of albumen by the kidneys, impotence, a form of delirium tremens different from that caused by alcoholic excesses, intermittent fever, and great derangement of the moral character, are among the morbid appearances produced.—*Med. and Surg. Reporter.*

---

*Female Physicians.*—The following facts, which are taken from *l' Avenir des Femmes*, a paper edited by M. Leon Richer, may prove interesting to our readers:

The Medical Faculty of Boston apparently deserve the credit of being the first to receive female students; this was in the year 1848. Zurich admitted its first female student in 1862; and in Russia, the Ecole de Medecine de Saint-Petersbourg was thrown open to women in 1872. In Germany, the Universities have only in rare instances granted diplomas to women. The first female to receive the diploma of Doctor of Medicine in that country was Mlle. von Siebold. Since 1874, Leipsic has graduated three female doctors. In Italy, the higher schools were only established in 1864, the municipality of Milan taking the initiative; and up to the present time only one woman has been granted a diploma. This was Mlle. Giraldi, who received the degree of Licentiate of Letters in Naples. In France, the first "bacheliere" (Mlle. Daubie) was received in 1861, and the female physician (Mde. Garret) in 1870. The University of London has been the most recalcitrant. It is only since the vote of the 15th of last January that women have been admitted to the examinations for all the diplomas, viz., those of medicine, arts, sciences, and law.—*The Medical Record.*

---

*On the Absorption of Lime Salts.*—The opinion of physicians concerning the therapeutic effect of lime salts differ widely. Some think that they act only *locally* upon the mucous membranes, the secretions and contents of the intestines, but that they have *no general* action upon the system Others consider them as powerful remedies in all so-called dyscrasies. These differences in opinion caused the author to experiment with animals, to see if lime salts *enter the system*, and become excreted with the urine. He found that soluble lime salts, when brought into the body, become *absorbed*, though only to a small degree. We may therefore expect that a deficit of lime in the body may be balanced by the introduction of a soluble lime salt in a sufficient quantity, provided the general condition of the body favors a return to the standard. The administration of these salts in the proper diseases seems therefore to be well founded.—*Dr. Perl, in Virchow's Arch.*

---

*The Skulls of Women.*—M. Lebon, in a communication made to the Congres d'Anthropologie in Paris, pointed out that, while the relative volume of the skull, compared with the rest of the skeleton, has increased with the progress of civilization, the difference in size between the skulls of men and women is also much less in the savage than among the civilized races. This difference was admitted by the ethnologists present, and was explained by the President, M. Broca, on the ground that among the primitive races women led much the same lives as men, and took an equal part in the struggle for existence. According to these anthropological data the "protection" of women and their exclusion from professional struggles has ended in lessening the cranial capacity, therefore presumably the brain-power.—*Medical and Surgical Reporter.*

*Healing of Pieces Separated from the Human Body.*—Two cases occurred in the clinic of Dr. Maas, in which pieces separated from the body were replaced, and had united. In both cases the nose was the portion injured, and in both the epidermis sloughed off, leaving the rete malpighii exposed. The pieces were sewed on with fine sutures, between which strips of adhesive plaster were applied, and, over all, oiled silk and cotton batting, to prevent evaporation and maintain warmth. The two following rules are drawn : 1. The piece separated must be kept warmed to the temperature of the human body. 2. It must be replaced, whether with sutures or adhesive plaster, or both, directly the flow of blood ceases. The nutrition of the piece is supposed to be maintained by the speedy re-establishment of circulation through its vessels.—*Cincinnati Lancet and Clinic.*

---

*Conservative Surgery.*—Dr. Borlee claims that a simple dressing of charpie and compresses wet with spirits of camphor, in connection with thorough drainage, deserves the preference over Lister's method. With this dressing erysipelas scarcely ever occurred, the suppuration, even when the wounds were extensive, was slight, and the secretion did not become offensive He illustrates the method by the histories of eight cases, comprising one case of "loose cartilage" in the knee joint, removed by excision, with perfect recovery, two cases of severe, complicated fracture of the leg, an amputation and an exarticulation of the upper extremity, and extirpation of tumors.—*Centralblatt f. Chirurgie.*

---

*Disadvantages of Salicylic Acid as a Mouth-Wash.*—Dr. Buch, of St. Petersburg, has found that even a very weak solution of salicylic acid is injurious to the teeth. After it has been used for some time, the teeth appear softer, and feel as if they were covered with some gritty substance. This is due to the acid combining with one or more of the constituents of the teeth, the surface of which becomes soft and granular. Dr. Buch believes that a salicylate of lime is formed, which adheres to the teeth, and is the cause of the peculiar sensation when they are rubbed together.—*Edinburgh Medical Journal.*

---

*Heavy Sentence of a Dentist*—The Court of Assizes of the Seine-Inferieure, France, recently condemned a dentist named Paul Levy to ten years' imprisonment for the crime of rape. His victim was a young girl, 20 years of age, and the crime was committed while she was under the influence of an anæsthetic. Levy is married and is the father of three children. He has practiced his profession for some time in the city of Lyons.—*Medical Record.*

